# Exploring College Men’s Human Papillomavirus (HPV) Vaccine Behavior and Intention in the United States

**DOI:** 10.1007/s10900-025-01489-z

**Published:** 2025-06-14

**Authors:** Olivia H. Marshall, Dayna S. Henry, Laura K. Merrell, James M. Bishop

**Affiliations:** Department of Health Sciences, James Madison University, 235 Martin Luther King Jr Way, Harrisonburg, VA 22807 USA

**Keywords:** Human papillomavirus, HPV, College men, Vaccine

## Abstract

The purpose of this study was to measure the HPV vaccine behaviors and intentions of college-aged men. 493 participants, who identified as “male” or “trans man”, from a large university in the Mid-Atlantic were included in this study. An online survey measured several independent variables as well as variables related to HPV and HPV vaccine knowledge, perceptions, and behaviors. Descriptive statistics were used to evaluate each variable after filtering the participants by vaccination status. A binomial logistic regression was used to analyze and predict the effect of each variable on college men’s vaccination status. The academic year in which participants completed the survey, home location, and HPV knowledge were significant predictors of HPV vaccination status. Lack of inclusion of males during conversations about the HPV vaccine could be the source of the differences between male and female HPV vaccination behavior and intention.

## Introduction

Human papillomavirus (HPV) is the most prevalent sexually transmitted infection in the United States and the most frequent viral infection of the reproductive system [1, 2]. Almost every sexually active person will become infected with HPV in their lifetime [3]. According to the CDC, there were approximately 43 million cases of HPV in 2018 [4]. Prevalence of HPV was 45.8% in adult males and 40.1% in adult females, both aged 14 through 59 years old [5]. Moreover, almost half of the new HPV infections each year are in people 15 through 24 years old, with people aged 20 through 24 years old having the highest prevalence at 62.8% in women and 41.8% in men [5, 6]. This highlights the increased risk for HPV posed to college-aged young adults.

The family of human papillomaviruses affect the skin and mucous membranes [7]. Transmission of HPV occurs through close skin-to-skin contact with an HPV-infected person, often through different types of sexual activity (oral, vaginal, penile, and anal sex); though not all HPV infections are transmitted sexually [8]. The main factors increasing risk of HPV infection are linked to sexual behavior and include a higher number of recent and overall sexual partners [8, 9]. Other factors such as a weakened immune system and coinfections with other sexually transmitted diseases also increase the likelihood of HPV infection [10]. Most people infected with HPV never experience symptoms and about 90% of HPV infections will subside within 2 years [3, 11]. Transmission of HPV is still possible even if an infected person has no symptoms [4].

There are over 200 different types of HPV: classified as low-risk or high-risk depending on their outcomes. Low-risk HPV infections can lead to benign cervical growths, warts on the anus or genitals, or wart-like growths on the respiratory tract [12, 13]. Out of all sexually active adults in the US, approximately one in 100 has a current genital wart infection [4]. Low-risk types of HPV can also cause common warts on the hands and face, as well as plantar warts on the feet. Both common and plantar warts are not commonly caused by sexually transmitted strains [14]. High-risk HPV infections, however, can lead to benign cervical growths, pre-cancerous cervical growths, and cancers of the oropharyngeal and anogenital regions. Cancers in the anogenital region include cancer of the anus, vagina, vulva, and penis [15]. High-risk HPV infections can cause cancer, but not all high-risk HPV infections lead to cancer [15, 16]. The CDC estimates that 36,000 cases of cancer in the United States are caused by HPV in both men and women every year [3]. The most common HPV-related cancer in women is cervical cancer and the most common HPV-related cancer in men is oropharyngeal cancer, which includes cancer of the throat, tongue, and tonsils [17].

There is a safe and effective primary prevention strategy for HPV through vaccination [4]. There is currently one vaccine to prevent HPV that is approved by the Food and Drug Administration (FDA) and marketed in the United States: Gardasil 9 [18, 19]. Gardasil 9 offers protection against seven types of high-risk HPV causing cancer (types 16, 18, 31, 33, 45, 52, and 58) and two types of low-risk HPV causing genital warts (types 6 and 11), totaling protection against 9 types of HPV [19, 20]. It is recommended children ages 9 through 14 receive two doses of the HPV vaccine, while people aged 15 through 26 should receive three doses [21]. Most people currently aged 18 through 26 with at least one dose of the vaccine were between 13 and 17 years old when they received their first dose [22]. In 2018, the FDA approved the use of Gardasil 9 for women and men aged 27 through 45 years old [19]. However, the CDC does not recommend HPV vaccination for everyone aged 27 through 45 because most sexually active adults have already been exposed to HPV, highlighting the need for vaccination before age 26 [4].

The introduction of the HPV vaccine has led to positive health outcomes relating to the prevalence of HPV cases. Receiving at least one dose of the HPV vaccine provides approximately 82% effectiveness in protection against HPV infection [23]. In 14 through 19-year-old women, the prevalence of high-risk types of HPV that can be prevented with the vaccine had a 56% decrease following introduction of the HPV vaccine in the United States [23]. There is little information available about possible decreases in HPV incidence or prevalence in males following vaccination.

The Healthy People 2020 goals for HPV vaccination were that 80% of women and men ages 13 through 15 to have received at least two doses of the HPV vaccine by 2020; however, in 2018, only 48% of adolescents nationwide had achieved this goal [24, 25]. In Virginia, 51.5% of adolescent women and 37.6% of adolescent men had received at least two doses of the HPV vaccine in 2018 [26, 27]. The updated Healthy People 2030 goals remain at increasing the proportion of HPV vaccinated adolescents to 80% [25].

Though the number of HPV-vaccinated young adults (ages 18 through 26) almost doubled between 2013 and 2018; only 39.9% of young adults had received at least one dose of the HPV vaccine, and only 21.5% of young adults had received the recommended number of doses in 2018 [22]. Approximately 50% of college students have received at least one dose of the HPV vaccine [28, 29]. Since the HPV vaccine is recommended through 26 years of age and available through 45 years of age (depending on healthcare provider recommendations), many unvaccinated college students are still eligible for vaccination [4].

Despite men having a higher prevalence of HPV infection than women, they consistently have lower HPV vaccination rates than women [5]. Male vaccination rates have consistently remained about 20% points below their female counterparts from 2013 to 2018 [22]. In addition, women ages 18 through 26 are more likely than men to have received at least one dose of the HPV vaccine or be fully vaccinated against HPV. Furthermore, whereas most women tend to have received their first dose of the HPV vaccine before they turned 13 years old, men are more likely to do so at a later age (18 through 26 years old) [22]. Moreover, one study reported that approximately 80% of unvaccinated male college students also did not intend to receive the HPV vaccine [30].

College students’ levels of knowledge about HPV and the HPV vaccine could account for the low levels of vaccine uptake in this age group. Most college students are aware of HPV and the HPV vaccine but have only a moderate level of HPV knowledge [28, 31, 32] with higher levels of knowledge being associated with those already vaccinated [28]. In addition, young adults with higher levels of knowledge on general health have more positive attitudes about the HPV vaccine than those with lower levels of knowledge (high school and college students) [33].

Differences between HPV prevalence in men and women could be due to gender differences in levels of knowledge about HPV. Studies measuring HPV knowledge in college men show about 30% of college men had little to no HPV knowledge, and about 45% of the participants did not know anything about the HPV vaccine prior to the study [34]. Unfortunately, in 2019, about two-thirds of college-aged men and half of college-aged women did not know the vaccine was recommended for people through 26-years-old [28]. Moreover, about 93% of college men do not believe they are not at risk for any sexually transmitted infections, even though most were sexually active and either never or rarely used condoms [35]. This highlights the inconsistencies in data found between genders, which is also seen across discussions surrounding HPV and the HPV vaccine.

In addition to knowledge, differences in HPV prevalence and vaccination uptake between men and women could also be influenced by parents and healthcare providers. Parents significantly influence young adults’ decisions to get vaccinated and many parents are hesitant about vaccinating their children against HPV [29]. There was widespread parental concern that HPV vaccination would increase sexual behavior, though multiple studies have found no relationship between HPV vaccination and increased sexual behavior [36, 37]. Receiving the HPV vaccine is significantly related to discussion and recommendation from a healthcare provider about the HPV vaccine [28]. Women were more likely than men to state their healthcare provider gave them information about HPV [31]. This could be one reason for reduced perception and knowledge about HPV in males and could account for the lower vaccination rates in men. Most college men are not aware the HPV vaccine is recommended for men [30]. There has been a consistent lack of understanding of the effect of HPV on men, as shown by the almost three-year lag between vaccination recommendations for women and men [38, 39]. Routine HPV vaccination was not recommended for men until 2011, almost five years after the HPV vaccine was licensed for routine use in women [40]. These factors could be the reason for the higher prevalence of HPV and lower HPV vaccination rates in college-aged men.

The gender differences in HPV knowledge, prevalence, and vaccination rates highlight the need for further research on HPV and the HPV vaccine among college men. Men’s vaccination rates, knowledge, and awareness fall short, while HPV infection rates remain higher in men making it crucial to identify variables influencing male college students’ intention to get vaccinated against HPV [22]. Therefore, the purpose of this study was to identify which predictor variables were associated with college men’s behavior and intention to get vaccinated against HPV. Specifically, the current study addressed the following research questions:

1. Does age, relationship status, political affiliation, survey wave (academic year in which the survey was completed), type of sexual education received, parent education level, current sexual behavior, urban vs. rural home, or HPV knowledge level affect HPV vaccination status?
2. Among those who have not received the HPV vaccine, how many intend to receive the vaccine?
3. Among those who have received the HPV vaccine, who decided they would receive the vaccine?
4. Among those who have received the HPV vaccine, how many doses did they receive?
5. Among those who have received the HPV vaccine, at what age did they receive their first dose?

## Materials and Methods

### Participants

Students at a large public university in the mid-Atlantic enrolled in a general education health class were offered a Qualtrics survey by participating professors in Fall and Spring semesters from 2019 to 2021. Participants had to be at least 18 years old to be included in the study. Of the participants who completed the survey, the majority were 18–19 years old (*n* = 338, 68.6%), single (*n* = 298, 60.4%), straight (*n* = 460, 93.3%), first year college students (*n* = 247, 50.1%), from Virginia (*n* = 377, 76.47%), white or Caucasian (*n* = 403, 81.74%), and Catholic (*n* = 165, 33.47%). Most participants stated they were single and not dating (61.07%, *n* = 298), received comprehensive sexuality education in high school (*n* = 245, 50.10%), and were from a suburban area (*n* = 345, 70.70%). About 40% of participants reported their mother (*n* = 202, 41.39%) and father (*n* = 201, 41.19%) had finished college. Most participants reported they were not engaging in oral (*n* = 252, 52.39%), vaginal (*n* = 259, 53.85%), or anal (*n* = 442,91.89%) sex. In the 2019–2020 academic year, 231 participants completed the survey (46.95%), while 261 participants completed the survey in the 2020–2021 academic year (53.05%). Additional demographic characteristics are shown in Table 1.

**Table 1:** Demographic characteristics

Characteristic	*n*	%
*Age (N = 493)*		
18–19	338	68.6
20–21	123	24.9
22–23	28	5.7
24+	4	0.8
*Race/Ethnicity (N = 522)*		
African American or Black	41	7.85
American Indian or Alaskan Native	7	1.34
Caucasian or White	403	77.2
East or South Asian	33	6.32
Native Hawaiian or Pacific Islander	3	0.57
More than one race	15	2.87
Other	15	2.87
Choose not to answer	5	0.96
*Political Affiliation (N = 489)*		
Republican	156	31.9
Democrat	106	21.7
Independent	70	14.3
Undecided/Don’t know/Don’t have one	120	24.5
Other	37	7.6
*Sexual Orientation (N = 493)*		
LGBTQIA+	33	6.7
Straight	460	93.3
*Relationship Status (N=493)*		
Single, not dating	298	61.1
In a committed relationship, but not living together	139	24.5
Dating multiple people (seeing people but not committed to one person)	41	8.4
Living with partner	7	1.4
Married/engaged	2	0.4
*Survey Wave (N=492)*		
2019–2020	231	46.9
2020–2021	261	53.1
*Sexuality Education (N=488)*		
Comprehensive Sexuality Education	245	50.1
Not sure, but they talked about STDs and pregnancy	154	31.5
Abstinence only/until marriage sexuality education	42	8.6
Did not receive any sexuality education in high school	23	4.7
Did not know which type of sexuality education they received in high school	24	4.9
*Mother’s Education Level (N=488))*		
Middle school	2	0.4
High school	50	10.3
Some college	62	12.7
Finished college	202	41.4
Some graduate classes	9	1.8
Completed graduate school	95	19.5
Professional Degree (J.D., M.D., D.O., etc.)	52	10.7
Terminal Degree (Ph.D.)	12	2.5
Not applicable or don’t know	4	0.8
*Father’s Education Level (N=488)*		
Middle school	2	0.4
High school	57	11.7
Some college	52	10.7
Finished college	201	41.2
Some graduate classes	10	2.0
Completed graduate school	87	17.8
Professional Degree (J.D., M.D., D.O., etc.)	13	2.7
Terminal Degree (Ph.D.)	60	12.3
Not applicable or don’t know	6	1.2
*Home Location (N=488)*		
Urban (city)	57	11.7
Rural (country)	86	17.6
Suburban (outside of city)	345	70.7
*Current Sexual Behavior– Oral Sex (N=481)*		
Yes	229	47.6
No	252	52.4
*Current Sexual Behavior– Vaginal Sex (N=481)*		
Yes	222	46.2
No	259	53.9
*Current Sexual Behavior– Anal Sex (N=481)*		
Yes	39	8.1
No	442	91.9

Of the 6,476 people that were offered the survey, 1,878 submitted the survey. The response rate for this survey was 28.99%. All responses were not included in the analysis due to the focus on male participants and elimination of participants who did not answer every question of interest. Participants that did not identify as “male” or “trans man” were eliminated due to the focus of this study (*n* = 1385). The remaining participants that identified as “male” or “trans man” were included and used in the data analysis (*n* = 493). Participants who did not fully answer the survey were kept in the dataset but eliminated from the analyses associated with the questions they skipped, resulting in different numbers of participants for some analyses.

### Procedures

Approval was given from the Institutional Review Board (IRB) and data collection began in Fall 2019. Data for this study were collected from Fall 2019 through Spring 2021 using two datasets, one from each academic year. Extra credit was given by the general education health class professors to students who completed the anonymous survey. Professors teaching the participating class sections posted the Qualtrics survey link in their course Canvas site for students to access and complete within a stated time period. The first page of the survey contained a consent cover letter that explained the study, benefits, risks, and contact information if they had any questions or concerns. Those who agreed to participate proceeded with the survey. Other extra credit opportunities were available for students who did not wish to participate.

### Measures

The larger survey assessed demographics, personality, gender discrimination, racial/ethnic discrimination, sexual behavior, alcohol, tobacco, and other drug use. Demographic questions measured age, sex, gender identity, sexual orientation, race, religion, and political identity. The survey also included questions on living arrangements, academic major, academic year, Greek life affiliation, type of sexual education received, parent education level, urban vs. rural home, and relationship status. Half of the class sections participating in the assessment were randomly selected to receive additional questions on online dating, cybervictimization, bystander behavior, and HPV.

This study focused on data from two academic years: 2019–2020 and 2020–2021, which were coded into the predictor variable, “survey wave”. Age was measured categorically with 4 answer choices: “18–19”, “20–21”, “22–23”, and “24+”. “24+” was excluded from the analysis due to a small number of responses. Relationship status was measured as “single, not dating”, “married/engaged”, “living with partner”, “dating multiple people (you are seeing people, but not committed to one person)”, and “in a committed relationship but not living with partner”. “Married/engaged” was excluded from the analysis due to a small number of responses. Two responses from 2020 to 2021 data set for the question “What is your current relationship status?” answered “other” and wrote in responses using the string text box. Using the participants’ answers, one response was recoded into the answer choice “In a committed relationship but not living together” and the other was recoded to “Dating multiple people (you are seeing people, but not committed to one person).” “Other” was not an answer choice for the 2019–2020 survey, so these responses were recoded to ensure the questions were identical across the datasets.

Political affiliation was measured as “Republican”, “Democrat”, “Independent”, “Libertarian”, “Socialist”, “undecided”, “don’t know”, and “don’t have one”. Type of sexuality education received was measured as “Comprehensive Sexuality Education”, “Abstinence Only/Until Marriage Education”, “none”, “not sure but we talked about sexually transmitted diseases and pregnancy”, and “I don’t know”. Parent education level was measured as “middle school”, “high school”, “some college”, “finished college”, “some graduate classes”, “completed graduate school”, “Professional Degree (J.D., M.D., D.O., etc.)”, “Terminal Degree (Ph.D.)”, “not applicable”, and “don’t know” for both parents. Current sexual behavior was measured as “yes” or “no” for currently engaging in oral, vaginal, and anal sex. Home location was measured as “urban (city)”, “rural (country)”, and “suburban (outside of city)”.

Knowledge about HPV was measured with 31 true/false questions from a validated instrument about HPV [41]. HPV knowledge questions were recoded from “true,” “false,” and “unsure” to “correct” and “incorrect.” A score of 0 was given for every incorrect answer and a score of 1 was given for every correct answer so the lowest possible score was 0 and the highest was 31 with higher scores indicating more HPV knowledge. The answer option “unsure” was considered “incorrect.” Answering “unsure” for a knowledge question was considered incorrect because the participants did not know the correct answer to these true or false questions, and therefore their answers of “unsure” were considered incorrect in this scenario. It is important to note a substantial number of participants who selected “unsure” for almost every HPV knowledge question. Participants who selected “unsure” ranged from 45.2% (*n* = 210) to 72.9% (*n* = 337) between the 31 HPV knowledge questions.

Other questions on HPV included, “Have you received the HPV vaccine (also known as Gardasil or Cervarix?” (yes/no). If the participant responded “yes,” they had received the vaccine, they were asked how many doses they received (one/two/three/unsure), how old they were when they received their first dose (11–12 years old/13–17 years old/18–26 years old), and who decided they would receive the HPV vaccine (themselves/a parent or guardian/joint decision/unsure). If the participant responded “no,” they had not received the vaccine, they were asked whether they intended to receive the HPV vaccine (yes/no/unsure). If they responded they did intend to receive the HPV vaccine, they were asked when they planned to receive it (within a month/8 months/a year/more than a year).

### Analysis

Statistical Package for the Social Science (SPSS) was used to analyze the data. Descriptive statistics were used to examine frequency data to describe the percentages and frequencies of all variables included in the study, except the HPV knowledge score, for which, mean, standard deviation, and range was described. Among those who were vaccinated, descriptive statistics were used to examine when and who decided they received the HPV vaccine. Among those who were not vaccinated, descriptive statistics were used to examine their intention to get vaccinated.

HPV vaccination status was predicted with a binomial logistic regression using survey wave, age, relationship status, political affiliation, type of sexual education received, parent education level, current sexual behavior, urban vs. rural home, and HPV knowledge as predictors. Participants who responded “unsure” to the HPV vaccination status question were eliminated from the analysis (*n* = 178). This was done to ensure validity of the results, as being “unsure” of vaccination status cannot be predicted. A dummy variable for HPV vaccination status was created using only the “yes” and “no” answer choices (0 = No, 1 = Yes). The dichotomous dependent variable was coded as 0 for the negative response (unvaccinated) and 1 for the positive response (vaccinated). Unvaccinated and vaccinated participants were assigned 0 and 1 respectively. The total number of participants included in the binomial logistic regression was 279 due to missing cases from each variable.

## Results

Overall, 44.9% of participants reported they had received the HPV vaccine (*n* = 216), 18.2% reported they had not received the HPV vaccine (*n* = 88) and 36.9% were unsure whether they had received the HPV vaccine (*n* = 178). The average score of the HPV knowledge scale was 22.90 (SD = 4.84) with scores ranging from 11 to 30.

### Descriptive Results Among Vaccinated Participants

Among those who had received the HPV vaccine (*n* = 216), almost half of the participants reported that a parent/guardian decided they would receive the HPV vaccine and almost a third stated it was a joint decision. Over one-third of participants stated they were unsure how many doses of the HPV vaccine they had received and over one-quarter reported they received 3 doses of the HPV vaccine. Over one-third of participants reported they were 13–17 years old when they received their first dose of the HPV vaccine. Another one-third of participants reported they were unsure what age they were when they received their first dose of the HPV vaccine. Descriptive statistics for those who did receive the HPV vaccine are fully shown in Table 2.

### Descriptive Results Among Unvaccinated Participants

Among those who have not received the HPV vaccine (*n* = 88), almost half of participants stated they were unsure if they intend to receive the HPV vaccine. Over one-third of participants stated they did not intend to receive the HPV vaccine. Among those who stated they did intend to receive the HPV vaccine, over half of participants stated they were unsure when they planned to receive their first dose of the HPV vaccine. Over one-third of participants who stated they did intend to receive the HPV vaccine stated they planned to receive their first dose of the HPV vaccine within the next month. Descriptive statistics for those who did not receive the HPV vaccine are fully shown in Table 2.

**Table 2:** Descriptive statistics among vaccinated versus unvaccinated participants

*Vaccinated (n = 216)*				
Who decided you would receive the HPV vaccine	Parent/Guardian *n* = 105, 48.61%	Joint decision *n* = 63, 29.17%	Themselves *n* = 36, 16.67%	Unsure *n* = 12, 5.56%
How many doses they received	1 dose *n* = 27, 12.50%	2 doses *n* = 46, 21.30%	3 doses *n* = 62, 28.70%	Unsure *n* = 81, 37.50%
How old they were when they received their first dose	11–12 years old *n* = 35, 16.10%	13–17 years old *n* = 76, 35.00%	18–26 years old *n* = 33, 15.20%	Unsure *n* = 73, 33.60%
*Unvaccinated (n = 88)*				
Whether they intended to receive the HPV vaccine	Yes *n* = 14, 15.90%	No *n* = 36, 40.9%	Unsure *n* = 38, 43.2%	
If they did intend to receive the HPV vaccine, when they planned to receive it	Within a month *n* = 5, 35.71%	Within a year *n* = 1, 7.14%	Unsure *n* = 8, 57.14%	

### Inferential Results

A binomial logistic regression was performed to determine how survey wave, age, relationship status, political affiliation, type of sex education received, parent education level, current sexual behavior, urban vs. rural home, and HPV knowledge predicted HPV vaccination status. The logistic regression model was statistically significant, c^2^(41) = 76.33, *p* < 0.001. The model explained 34.0% (Nagelkerke R^2^) of the variance in HPV vaccination status and correctly classified 77.8% of cases. Sensitivity was 92.3%, specificity was 43.4%, positive predictive value was 79.4% and negative predictive value was 70.6%. The area under the ROC curve was 0.801, 95% CI [0.746, 0.857]. Out of the ten variables, three of the variables were statistically significant: survey wave, home location, and HPV knowledge score (as shown in Table 3). Students in the 2019–2020 academic year had 0.530 smaller odds of being vaccinated for HPV versus students in the 2020–2021 academic year (*p* < 0.05). Students who described their home location as “urban (city),” rather than rural or suburban, had a higher likelihood of being vaccinated for HPV (*p* < 0.05). Higher HPV knowledge scores are associated with an increased likelihood of having received the HPV vaccine (*p* < 0.05).

**Table 3:** Logistic regression predicting likelihood of HPV vaccination based on survey wave, age, relationship status, political affiliation, type of sexual education received, parent education level, current sexual behavior, urban vs. rural home, and HPV knowledge

	B	SE	Wald	df	*p*	Odds Ratio	95% CI for Odds Ratio	
							Lower	Upper
*Survey Wave*^*a*^								
2019–2020								
2020–2021	− 0.635	0.324	3.842	1	0.05	0.530	0.281	1.000
*Age*^b^								
18–19								
20–21	− 0.640	0.384	2.776	1	0.096	0.527	0.249	1.119
22–23	− 0.838	0.752	1.241	1	0.265	0.433	0.099	1.890
*Relationship Status*^c^								
Single, not dating								
Living with partner	− 0.107	1.373	0.006	1	0.938	0.899	0.061	13.250
Dating multiple people	0.859	0.638	1.813	1	0.178	2.360	0.676	8.238
In a committed relationship, but not living together	0.297	0.429	0.481	1	0.488	1.346	0.581	3.118
*Political Affiliation*^d^								
Republican Democrat	-0.179	0.474	0.143	1	0.705	0.836	0.330	2.116
Independent	0.269	0.531	0.256	1	0.613	1.308	0.462	3.707
Libertarian	1.070	0.937	1.303	1	0.254	2.915	0.465	18.287
Socialist	0.082	0.800	0.011	1	0.918	1.085	0.226	5.203
Undecided	− 0.752	0.598	1.585	1	0.208	0.471	0.146	1.520
Don’t know	0.030	0.744	0.002	1	0.968	1.030	0.239	4.431
Don’t have one	− 0.609	0.600	1.031	1	0.310	0.544	0.168	1.763
*Type of Sexual Education Received*^e^								
Comprehensive Sexuality Education								
Abstinence Only/Until Marriage Education	− 0.143	0.682	0.044	1	0.833	0.866	0.228	3.299
None	− 0.486	0.882	0.304	1	0.582	0.615	0.109	3.465
Not sure, but we talked about sexually transmitted diseases and pregnancy	− 0.226	0.374	0.367	1	0.545	0.797	0.383	1.659
I don’t know	− 0.110	0.779	0.020	1	0.888	0.896	0.194	4.126
*Father Education Level*^f^								
Middle school								
High school	1.315	1.043	1.590	1	0.207	3.723	0.482	28.739
Some college	1.019	0.857	1.414	1	0.234	2.771	0.516	14.876
Finished college	−19.187	14481.390	0.000	1	0.999	0.000	0.000	
Some graduate classes	0.664	0.907	0.537	1	0.464	1.943	0.328	11.499
Completed graduate school	0.554	1.158	0.229	1	0.632	1.741	0.180	16.839
Professional Degree (J.D., M.D., D.O., etc.)	0.650	1.508	0.186	1	0.667	1.915	0.100	36.770
Terminal Degree (Ph.D.)	20.236	40192.969	0.000	1	1.000		0.000	
*Mother Education Level*^g^								
Middle school								
High school	− 0.420	0.692	0.368	1	0.544	0.657	0.169	2.551
Some college	0.554	0.587	0.891	1	0.345	1.740	0.551	5.494
Finished college	1.650	1.425	1.342	1	0.247	5.210	0.319	85.019
Some graduate classed	0.692	0.613	1.278	1	0.258	1.999	0.602	6.641
Completed graduate school	− 0.089	0.724	0.015	1	0.903	0.915	0.221	3.784
Professional Degree (J.D., M.D., D.O., etc.)	21.754	14255.628	0.000	1	0.999		0.000	
Terminal Degree (Ph.D.)	−1.481	56841.855	0.000	1	1.000	0.227	0.000	
*Current Sexual Behavior– Oral Sex*^h^								
Yes								
No	0.641	0.838	0.585	1	0.444	1.899	0.367	9.821
*Current Sexual Behavior– Vaginal Sex*^i^								
Yes								
No	− 0.930	0.832	1.251	1	0.263	0.394	0.077	2.013
*Current Sexual Behavior– Anal Sex*^j^								
Yes								
No	1.062	0.691	2.363	1	0.124	2.893	0.747	11.209
*Home Location*^k^								
Urban (city)								
Rural (country)	1.306	0.645	4.109	1	0.043	3.693	1.044	13.062
Suburban (outside of city)	0.811	0.508	2.551	1	0.110	2.251	0.832	6.091
*HPV Knowledge*								
Cumulative Score	0.066	0.020	11.334	1	0.001	1.068	1.028	1.110

^a^Survey Wave was represented by 1 dummy variable with 2019–2020 serving as the reference group

^b^Age was represented by 2 dummy variables with 18–19 serving as the reference group

^c^Relationship Status was represented by 4 dummy variables with Single, not dating serving as the reference group

^d^Political Affiliation was represented by 7 dummy variables with Republican serving as the reference group

^e^Type of Sexual Education Received was represented by 4 dummy variables with Comprehensive Sexuality Education serving as the reference group

^f^Father Education Level was represented by 7 dummy variables with Middle School serving as the reference group

^g^Mother Education Level was represented by 7 dummy variables with Middle School serving as the reference group

^h^Current Sexual Behavior– Oral Sex was represented by 1 dummy variable with Yes serving as the reference group

^I^Current Sexual Behavior– Vaginal Sex was represented by 1 dummy variable with Yes serving as the reference group

^j^Current Sexual Behavior– Anal Sex was represented by 1 dummy variable with Yes serving as the reference group

^k^Home Location was represented by 2 dummy variables with Urban (city) serving as the reference group

## Discussion

The purpose of this study was to measure the HPV knowledge, vaccine behaviors and intentions of college-aged men at a large public university in the mid-Atlantic. About half of participants were vaccinated against HPV (*n* = 217, 44.9%). A vaccination rate of approximately 50% is consistent with other findings on college students’ vaccination rates, as well as male college student vaccination rates, specifically seen in Lee and colleagues [28, 29, 42]. The average HPV knowledge score was 22.90 (SD = 4.84), using a scale from 0 to 31. This is also consistent with previous literature stating college students have moderate levels of HPV knowledge [28, 31, 32].

Participants in the 2020–2021 academic year had a higher likelihood of having received the HPV vaccine compared to students in the 2019–2020 academic year. Living in an urban area increased the likelihood of receiving the HPV vaccine, compared to living in a rural or suburban area. People with higher HPV knowledge were more likely to be vaccinated against HPV.

The differences in HPV vaccination rates between students in the 2019–2020 academic year versus the 2020–2021 academic year could be due to the changes in vaccination recommendations for men around the time the participants would have been eligible for vaccination as adolescents. The HPV vaccine Gardasil was not licensed for use in men (ages 9 through 26) until 2009– three years after it was licensed for use in women [38, 39]. The Advisory Committee on Immunization Practices licensed the vaccine for men to prevent genital warts and did not recommend routine HPV vaccination for men at the time [38]. The Advisory Committee on Immunization Practices first recommended routine HPV vaccination in men in 2011, after reviewing factors such as HPV-related disease and cancer estimates [40]. Therefore, there was a 5-year gap between routine vaccine recommendation between women and men. This gap between male and female recommendations could account for the differences between vaccination rates between participants in the two academic years. This time period in which HPV vaccination was recommended for males is around the time participants in the later academic year (2020–2021) would be entering the age range in which HPV vaccination was recommended, resulting in a higher rate of HPV vaccination in men in the 2020–2021 academic year.

Those living in an urban area were more likely to have received the HPV vaccine than those living in a rural area. This is consistent with other studies who have found HPV vaccination was lower among adolescents from rural communities compared to those from urban communities [43]. Living in an urban area increasing the likelihood of receiving the HPV vaccine could be due to the higher availability of healthcare providers and clinics in which to obtain the vaccine. Those living in rural communities may not have as much access to preventive medicine as people living in urban communities. Differences in socioeconomic status between those living in rural areas versus those living in urban areas could also be a confounding variable to these findings. Incorporating alternative sites for HPV vaccination in underserved areas, such as in schools, would aid in expanding HPV vaccination rates in these communities [44].

A higher level of HPV knowledge being associated with HPV vaccination is consistent with the literature [28]. Higher levels of knowledge and level of education has been associated with HPV vaccination [33]. The finding that a higher level of HPV knowledge increasing the likelihood of being vaccinated for HPV is an expected addition to the previous literature that found comparable results; however, no other literature focused on HPV knowledge predicting HPV vaccination in college men specifically [28]. It is important to mention the significant number of participants that selected “unsure” for a large number of the HPV knowledge questions, which were ultimately coded as “incorrect” due to the participants not knowing the correct answers. The high number of participants who were unsure about most HPV knowledge questions could be explained by the relatively low levels of health literacy in college students [45, 46].

Among those who were vaccinated against HPV, most stated their parent or guardian decided they would receive the HPV vaccine. Other studies have showed equivalent results that parents significantly affect young adults’ decision to get vaccinated [29]. Among those who had received the HPV vaccine, most were unsure how many doses they received. Other studies have also found that many of their participants were unsure of their vaccination status or how many doses they received [47]. Being unsure of how many doses received could be due to the finding that parents/guardians were the most likely to decide they would receive the HPV vaccine. Additionally, since women are more likely than men to receive information about HPV from their healthcare provider, men could have not been informed about the HPV vaccine or their HPV vaccine status from their healthcare provider [31]. Among those who were vaccinated against HPV, most were 13–17 years old when they received the HPV vaccine. Other literature has also found most young adults (aged 18 through 26) with least one dose of the HPV vaccine were between 13 and 17 years old when they received their first dose [22]. Among those who had not received the HPV vaccine, almost half were unsure if they intended to receive the HPV vaccine and only 15% stated they did intend to get vaccinated. Additionally, among those who were not vaccinated against HPV and intended to receive the HPV vaccine, most were unsure when they intended to get the vaccine. This intention rate is similar to previous literature that found 70–80% of unvaccinated college men did not intend to get vaccinated against HPV [30, 48]. About one-third of unvaccinated participants who did intend to receive the HPV vaccine stated they planned to do so within the next month. These results could be explained by participants choosing the socially desirable answer or due to college students being given new freedoms and abilities to make their own medical decisions.

### Limitations

The limitations of this study include an undiversified sample, a small sample size due to incompletion of the survey, and an exclusion of responses for one variable due to the answer choice. The sample for this study was not demographically diverse due to the population from which it was sampled. Additionally, there was a small sample size for this study as many survey responses could not be included due to incompleteness. A significant number of participants did not answer every HPV knowledge question. Because the HPV knowledge variable was analyzed using a combined score of each 31 questions, these responses were excluded from the data analysis, and, therefore, lowered the sample size for analyses of HPV knowledge. Additionally, the substantial number of participants who selected “unsure” for each HPV knowledge question has implications for the results of this study. Because being “unsure” about a topic cannot be predicted, “unsure” was considered “incorrect” for the true or false HPV knowledge questions. Increasing the sample sizes for future research would contribute to the reliability of the findings.

### Suggestions for Future Research

A more diverse sample should be sought out so that that the effect of other demographic characteristics, such as ethnicity, religion, socioeconomic status, and sexual orientation, on HPV vaccination can be examined. Literature has shown that these factors can affect HPV vaccine uptake and attitudes in many populations, such as women [48–50], men that are not college students [51], and parents deciding to vaccinate their children [52, 53]. However, research has not focused on the influence of these factors on American college men specifically. In addition, due to the limitations discussed above, future research should utilize larger sample sizes to confirm the reliability of these findings. Future research utilizing these factors would help to identify areas in which to focus solutions to increase the HPV vaccination rates.

### Conclusion

HPV vaccination in college men was found to be significantly impacted by the academic year in which they responded to the survey, home location, and levels of knowledge about HPV. The lack of recommendation and education about HPV from healthcare providers to men could account for low male vaccination rates and HPV knowledge compared to women [31]. Lack of access to HPV education and vaccination in underserved areas could be another contributor to the gaps in HPV knowledge and vaccination. HPV vaccination clinics, HPV education in schools, and healthcare provider recommendations for everyone in the HPV vaccination age range to get vaccinated could all help improve the vaccination rates in men and close the gap between genders in HPV vaccination, awareness, and knowledge. Increasing vaccination rates in male college populations could lead to fewer negative health outcomes, including decreased HPV prevalence and lower HPV-related cancer rates. College-age males tend to have more non-monogamous sexual partners and encounters than females, which means they are most likely contributing to the spread of HPV [54, 55].

The results of this study have implications for practicing college health professionals, such as college health centers and healthcare providers that focus on college populations. With about half of college men being unvaccinated for HPV, it is important for college health centers and healthcare providers for college-age men to be aware of this statistic and provide additional education and encouragement to their college-age male patients about the importance of receiving the HPV vaccine. The HPV vaccine can potentially prevent more than 90% of HPV-related cancers [15]. Increasing HPV vaccination rates, especially in college male populations, is a safe and effective prevention measure to lower the risk of oropharyngeal and anogenital cancers.

## Data Availability

Not applicable.

## References

[1] Centers for Disease Control and Prevention (2023). Sexually transmitted diseases (STDs): Diseases & related conditions. https://www.cdc.gov/std/general/default.htm Accessed June 27, Updated March 2, 2023.

[2] World Health Organization (2022). Human papillomavirus (HPV) and cervical cancer. https://www.who.int/news-room/fact-sheets/detail/human-papillomavirus-(hpv)-and-cervicalcancer Published February 22, 2022. Accessed April 5.

[3] Centers for Disease Control and Prevention (2023). About HPV. https://www.cdc.gov/hpv/parents/about-hpv.html Updated February 10, Accessed June 27, 2023.

[4] Centers for Disease Control and Prevention (2022). Genital HPV infection– Basic fact sheet. https://www.cdc.gov/std/hpv/stdfact-hpv.htm Updated April 12, Accessed June 27, 2023.

[5] Lewis, R. M., Markowitz, L. E., Gargano, J. W., Steinau, M., & Unger, E. R. (2018). Prevalence of genital human papillomavirus among sexually experienced males and females aged 14–59 years, united states, 2013–2014. *The Journal of Infectious Diseases*, *217*(6), 869–877. 10.1093/infdis/jix655 29294016 10.1093/infdis/jix655 PMC5991084

[6] Satterwhite, C. L., Torrone, E., Meites, E., et al. (2013). Sexually transmitted infections among US women and men. *Sexually Transmitted Diseases*, *40*(3), 187–193. 10.1097/olq.0b013e318286bb53 23403598 10.1097/OLQ.0b013e318286bb53

[7] Gheit, T. (2019). Mucosal and cutaneous human papillomavirus infections and Cancer biology. *Frontiers in Oncology*, *9*, 355. 10.3389/fonc.2019.00355 31134154 10.3389/fonc.2019.00355 PMC6517478

[8] Veldhuijzen, N. J., Snijders, P. J., Reiss, P., Meijer, C. J., & van de Wijgert, J. H. (2010). Factors affecting transmission of mucosal human papillomavirus. *The Lancet Infectious Diseases*, *10*(12), 862–874. 10.1016/s1473-3099(10)70190-0 21075056 10.1016/S1473-3099(10)70190-0

[9] Itarat, Y., Kietpeerakool, C., Jampathong, N., et al. (2019). Sexual behavior and infection with cervical human papillomavirus types 16 and 18. *International Journal of Women’s Health*, *11*, 489–494. 10.2147/ijwh.s218441 31692583 10.2147/IJWH.S218441 PMC6716589

[10] Liu, G., Sharma, M., Tan, N., & Barnabas, R. V. (2018). HIV-positive women have higher risk of human papilloma virus infection, precancerous lesions, and cervical cancer. *Aids (London, England)*, *32*(6), 795–808. 10.1097/qad.0000000000001765 29369827 10.1097/QAD.0000000000001765 PMC5854529

[11] Rodriguez, A. C., Schiffman, M., Herrero, R., et al. (2008). Rapid clearance of human papillomavirus and implications for clinical focus on persistent infections. *Journal of the National Cancer Institute*, *100*(7), 513–517. 10.1093/jnci/djn044 18364507 10.1093/jnci/djn044 PMC3705579

[12] Doorbar, J., Egawa, N., Griffin, H., Kranjec, C., & Murakami, I. (2015). Human papillomavirus molecular biology and disease association. *Reviews in Medical Virology*, *25*(1), 2–23. 10.1002/rmv.1822 25752814 10.1002/rmv.1822 PMC5024016

[13] Egawa, N., & Doorbar, J. (2017). The low-risk papillomaviruses. *Virus Research*, *231*, 119–127. 10.1016/j.virusres.2016.12.017 28040475 10.1016/j.virusres.2016.12.017

[14] de Koning, M. N. C., ter Schegget, J., Eekhof, J., et al. (2010). Evaluation of a novel Broad-Spectrum PCR-Multiplex genotyping assay for identification of cutaneous Wart-Associated human papillomavirus types. *Journal of Clinical Microbiology*, *48*(5), 1706–1711. 10.1128/jcm.02122-09 20237103 10.1128/JCM.02122-09 PMC2863857

[15] Senkomago, V., Henley, S. J., Thomas, C. C., Mix, J. M., Markowitz, L. E., & Saraiya, M. (2019). Human Papillomavirus–Attributable Cancers — United states, 2012–2016. *MMWR Morbidity and Mortality Weekly Report*, *68*(33), 724–728. 10.15585/mmwr.mm6833a3 31437140 10.15585/mmwr.mm6833a3 PMC6705893

[16] Muñoz, N., Bosch, F. X., de Sanjosé, S., et al. (2003). Epidemiologic classification of human papillomavirus types associated with cervical Cancer. *New England Journal of Medicine*, *348*(6), 518–527. 10.1056/nejmoa021641 12571259 10.1056/NEJMoa021641

[17] Centers for Disease Control and Prevention (2022). HPV-Associated cancer statistics. https://www.cdc.gov/cancer/hpv/statistics/index.htm Updated October 3, Accessed June 27, 2023.

[18] Centers for Disease Control and Prevention (2021). HPV vaccination is safe and effective. https://www.cdc.gov/hpv/parents/vaccinesafety.html Updated July 23, Accessed April 5, 2022.

[19] United States Food and Drug Administration [FDA] (2018). FDA approves expanded use of Gardasil 9 to include individuals 27 through 45 years old. https://www.fda.gov/news-events/pressannouncements/fda-approves-expanded-use-gardasil-9-include-individuals-27-through-45-yearsold Published October 5, Accessed April 5, 2022.

[20] National Cancer Institute (2023). HPV and cancer. https://www.cancer.gov/about-cancer/causesprevention/risk/infectious-agents/hpv-and-cancer Updated April 4, Accessed June 27, 2023.

[21] Centers for Disease Control and Prevention (2021). Human papillomavirus (HPV): Vaccine schedule and dosing. https://www.cdc.gov/hpv/hcp/schedules-recommendations.html Updated November 1, Accessed April 5, 2022.

[22] Boersma, P., & Black, L. I. (2020). Human papillomavirus vaccination among adults aged 18–26, 2013–2018. Centers for Disease Control and Prevention. https://www.cdc.gov/nchs/data/databriefs/db354-h.pdf. Published December 9, Accessed April 5, 2022.

[23] Markowitz, L. E., Hariri, S., Lin, C., et al. (2013). Reduction in human papillomavirus (HPV) prevalence among young women following HPV vaccine introduction in the united states, National health and nutrition examination surveys, 2003–2010. *The Journal of Infectious Diseases*, *208*(3), 385–393. 10.1093/infdis/jit192 23785124 10.1093/infdis/jit192

[24] Office of Disease Prevention and Health Promotion (2022). Healthy People 2020: Immunization and infectious diseases. https://www.healthypeople.gov/2020/topics-objectives/topic/immunizationand-infectious-diseases/objectives Updated February 6, Accessed April 5, 2023.

[25] Office of Disease Prevention and Health Promotion. Healthy People (2030).: Increase the proportion of adolescents who get recommended doses of the HPV vaccine– 11D-08. https://health.gov/healthypeople/objectives-and-data/browse-objectives/vaccination/increaseproportion-adolescents-who-get-recommended-doses-hpv-vaccine-iid-08 Published 2021. Accessed April 5, 2022.

[26] Office of Disease Prevention and Health Promotion. Healthy People (2020).: State-level data: Female adolescents receiving 2 or 3 doses of HPV vaccine by age 13–15. https://www.healthypeople.gov/2020/data/map/4657?year=2018 Updated October 27, 2021. Accessed April 5, 2022.

[27] Office of Disease Prevention and Health Promotion. Healthy People (2020).: State-level data: Male adolescents receiving 2 or 3 doses of HPV vaccine by age 13–15. https://www.healthypeople.gov/2020/data/map/10676?year=2018 Updated October 27, 2021. Accessed April 5, 2022.

[28] Kellogg, C., Shu, J., Arroyo, A., et al. (2019). A significant portion of college students are not aware of HPV disease and HPV vaccine recommendations. *Human Vaccines & Immunotherapeutics*, *15*(7–8), 1760–1766. 10.1080/21645515.2019.1627819 31166148 10.1080/21645515.2019.1627819 PMC6746478

[29] LaJoie, A. S., Kerr, J. C., Clover, R. D., & Harper, D. M. (2018). Influencers and preference predictors of HPV vaccine uptake among US male and female young adult college students. *Papillomavirus Research*, *5*, 114–121. 10.1016/j.pvr.2018.03.007 29578098 10.1016/j.pvr.2018.03.007 PMC5886909

[30] Hunter, T., & Weinstein, M. (2015). Beliefs and knowledge about the human papillomavirus vaccine among undergraduate men. *Health Education Journal*, *75*(2), 249–256. 10.1177/0017896915572705

[31] Kasymova, S., Harrison, S. E., & Pascal, C. (2019). Knowledge and awareness of human papillomavirus among college students in South Carolina. *Infectious Diseases*, *12*, 1–9. 10.1177/1178633718825077 10.1177/1178633718825077 PMC6351721 30728723

[32] Koplas, P. A., Braswell, J., & Saray Smalls, T. (2019). Uptake of HPV vaccine in traditional-age undergraduate students: Knowledge, behaviors, and barriers. *Journal of American College Health*, *67*(8), 762–771. 10.1080/07448481.2018.1512499 30395793 10.1080/07448481.2018.1512499

[33] Suryadevara, M., Bonville, J. R., Kline, R. M., et al. (2016). Student HPV vaccine attitudes and vaccine completion by education level. *Human Vaccines & Immunotherapeutics*, *12*(6), 1491–1497. 10.1080/21645515.2015.1123359 26836052 10.1080/21645515.2015.1123359 PMC4964676

[34] Catalano, H. P., Knowlden, A. P., Birch, D. A., Leeper, J. D., Paschal, A. M., & Usdan, S. L. (2016). Using the theory of planned behavior to predict HPV vaccination intentions of college men. *Journal of American College Health*, *65*(3), 197–207. 10.1080/07448481.2016.1269771 27960609 10.1080/07448481.2016.1269771

[35] Fontenot, H. B., Collins Fantasia, H., Charyk, A., & Sutherland, M. A. (2014). Human papillomavirus (HPV) risk factors, vaccination patterns, and vaccine perceptions among a sample of male college students. *Journal of American College Health*, *62*(3), 186–192. 10.1080/07448481.2013.872649 24328855 10.1080/07448481.2013.872649

[36] Brouwer, A. F., Delinger, R. L., Eisenberg, M. C., Campredon, L. P., Walline, H. M., Carey, T. E., & Meza, R. (2019). HPV vaccination has not increased sexual activity or accelerated sexual debut in a college-aged cohort of men and women. *Bmc Public Health*, *19*(1), 821. 10.1186/s12889-019-7134-1 31238911 10.1186/s12889-019-7134-1 PMC6593582

[37] Ratanasiripong, N. T. (2014). Human papillomavirus vaccine increases High-Risk sexual behaviors. *The Journal of School Nursing*, *30*(6), 456–463. 10.1177/1059840513520042 24414172 10.1177/1059840513520042

[38] Centers for Disease Control and Prevention (2010). FDA Licensure of Quadrivalent Human Papillomavirus Vaccine (HPV4, Gardasil) for Use in Males and Guidance from the Advisory Committee on Immunization Practices (ACIP). https://www.cdc.gov/mmwr/preview/mmwrhtml/mm5920a5.htm Updated May 28, Accessed April 5, 2022. 20508594

[39] Centers for Disease Control and Prevention (2010). Morbidity and Mortality Weekly Report (MMWR): FDA licensure of bivalent human papillomavirus vaccine (HPV2, Cervarix) for use in females and updated HPV vaccination recommendations from the Advisory Committee on Immunization Practices. https://www.cdc.gov/mmwr/preview/mmwrhtml/mm5920a4.htm Updated May 28, Accessed April 5, 2022. 20508593

[40] Centers for Disease Control and Prevention. Morbidity and Mortality Weekly Report (MMWR): Recommendation on the use of quadrivalent human papillomavirus vaccine in males - Advisory Committee on Immunization Practices (ACIP) (2011). https://www.cdc.gov/mmwr/preview/mmwrhtml/mm6050a3.htm Updated December 23, 2011. Accessed April 5, 2022. 22189893

[41] Daley, E. M., Vamos, C. A., Buhi, E. R., et al. (2010). Influences on human papillomavirus vaccination status among female college students. *Journal of Women’s Health*, *19*(10), 1885–1891. 10.1089/jwh.2009.1861 20815737 10.1089/jwh.2009.1861

[42] Lee, H. Y., Lust, K., Vang, S., & Desai, J. (2018). Male undergraduates’ HPV vaccination behavior: Implications for achieving HPV-Associated Cancer equity. *Journal of Community Health*, *43*(3), 459–466. 10.1007/s10900-018-0482-4 29470802 10.1007/s10900-018-0482-4

[43] Swiecki-Sikora, A. L., Henry, K. A., & Kepka, D. (2019). HPV vaccination coverage among US teens across the Rural‐Urban continuum. *The Journal of Rural Health*, *35*(4), 506–517. 10.1111/jrh.12353 30703854 10.1111/jrh.12353 PMC6669111

[44] Shah, P. D., Gilkey, M. B., Pepper, J. K., Gottlieb, S. L., & Brewer, N. T. (2014). Promising alternative settings for HPV vaccination of US adolescents. *Expert Review of Vaccines*, *13*(2), 235–246. 10.1586/14760584.2013.871204 24405401 10.1586/14760584.2013.871204 PMC4267674

[45] Dolezel, D., Shanmugam, R., & Morrison, E. E. (2018). Are college students health literate? *Journal of American College Health*, *68*(3), 242–249. 10.1080/07448481.2018.1539001 30457454 10.1080/07448481.2018.1539001

[46] Ickes, M. J., & Cottrell, R. (2010). Health literacy in college students. *Journal of American College Health*, *58*(5), 491–498. 10.1080/07448481003599104 20304761 10.1080/07448481003599104

[47] McLendon, L., Puckett, J., Green, C., et al. (2021). Factors associated with HPV vaccination initiation among united States college students. *Human Vaccines & Immunotherapeutics*, *17*(4), 1033–1043. 10.1080/21645515.2020.1847583 33325794 10.1080/21645515.2020.1847583 PMC8018405

[48] Cooper, D. L., Zellner-Lawrence, T., Mubasher, M., Banerjee, A., Hernandez, N. D., Examining, H. P. V., & Awareness (2018). Sexual behavior, and intent to receive the HPV vaccine among racial/ethnic male college students 18–27 years. *American Journal of Men’s Health*, *12*(6), 1966–1975. 10.1177/1557988318803163 30334489 10.1177/1557988318803163 PMC6199446

[49] Boakye, E. A., Osazuwa-Peters, N., López, J., Pham, V. T., Tobo, B. B., Wan, L., Schootman, M., & McElroy, J. A. (2021). Disparities in human papillomavirus (HPV) vaccine initiation and completion based on sexual orientation among women in the united States. *Human Vaccines & Immunotherapeutics*, *17*(2), 428–433. 10.1080/21645515.2020.1778407 32701386 10.1080/21645515.2020.1778407 PMC7899676

[50] Daniel-Ulloa, J., Gilbert, P. A., & Parker, E. A. (2016). Human papillomavirus vaccination in the united states: Uneven uptake by gender, race/ethnicity, and sexual orientation. *American Journal of Public Health*, *106*(4), 746–747. 10.2105/ajph.2015.303039 26890185 10.2105/AJPH.2015.303039 PMC4816000

[51] Boakye, E. A., Zeng, W., Governor, S., Nagendra, S., Tobo, B. B., Simpson, M. C., & Osazuwa-Peters, N. (2019). Differences in human papillomavirus (HPV) vaccine uptake by nativity status among men aged 18–34 years. *Preventive Medicine Reports*, *16*, 101010. 10.1016/j.pmedr.2019.101010 31799106 10.1016/j.pmedr.2019.101010 PMC6883324

[52] Shelton, R. C., Snavely, A. C., De Jesus, M., Othus, M. D., & Allen, J. D. (2013). HPV vaccine Decision-Making and acceptance: Does religion play a role?? *Journal of Religion and Health*, *52*(4), 1120–1130. 10.1007/s10943-011-9553-x 22076049 10.1007/s10943-011-9553-x PMC4616263

[53] Szilagyi, P. G., Albertin, C. S., Gurfinkel, D., et al. (2020). Prevalence and characteristics of HPV vaccine hesitancy among parents of adolescents across the US. *Vaccine*, *38*(38), 6027–6037. 10.1016/j.vaccine.2020.06.074 32758380 10.1016/j.vaccine.2020.06.074 PMC9495911

[54] Grello, C. M., Welsh, D. P., & Harper, M. S. (2006). No strings attached: The nature of casual sex in college students. *Journal of Sex Research*, *43*(3), 255–267. 10.1080/00224490609552324 17599248 10.1080/00224490609552324

[55] Vasilenko, S. A., & Lanza, S. T. (2014). Predictors of multiple sexual partners from adolescence through young adulthood. *Journal of Adolescent Health*, *55*(4), 491–497. 10.1016/j.jadohealth.2013.12.025 10.1016/j.jadohealth.2013.12.025 PMC4139487 24561033

